# The association between protection motivation and hepatitis b vaccination intention among migrant workers in Tianjin, China: a cross-sectional study

**DOI:** 10.1186/s12889-020-09292-2

**Published:** 2020-08-10

**Authors:** Cai Liu, Stephen Nicholas, Jian Wang

**Affiliations:** 1 School of Management, Tianjin University of Traditional Chinese Medicine, Tianjin, 301617 China; 2 Australian National Institute of Management and Commerce, 1 Central Avenue Australian Technology Park, Eveleigh Sydney, NSW 2015 Australia; 3 Research Institute for International Strategies, Guangdong University of Foreign Studies, Baiyun Avenue North, Guangzhou, 510420 People’s Republic of China; 4 School of Economics and School of Management, Tianjin Normal University, West Bin Shui Avenue, Tianjin, 300074 China; 5 Newcastle Business School, University of Newcastle, University Drive, Newcastle, NSW 2308 Australia; 6 Dong Fureng Institute of Economic and Social Development, Wuhan University, No.54 Dongsi Lishi Hutong, Dongcheng District, Beijing, 100010 China; 7 Center for Health Economics and Management at School of Economics and Management, Wuhan University, 299 Bayi Road, Wuchang District, Wuhan, 430072 Hubei Province China

**Keywords:** Migrant workers, Hepatitis b vaccination, Vaccination intention, Protection motivation theory, Health education

## Abstract

**Background:**

Migrant workers are a susceptible population to the hepatitis b virus (HBV) and a vulnerable spot in China’s immunization procedures. There is no free HBV immunization program for migrant workers in China, so understanding migrant workers’ motivation to receive the HBV vaccine is the first step in designing effective immunization policies.

**Methods:**

A fully specified protection motivation theory (PMT) model of HBV vaccination intention among migrant workers was specified. Data were collected through a cross-sectional survey of 406 migrant workers in three migrant-dense industries in Tianjin, China. Principal component factor analysis was used to produce PMT factors and nested binary logistic regression modeling was applied to assess the associations between protection motivation and HBV vaccination intention of migrant workers.

**Results:**

The nested binary logistic regression model suggested that the severity factor and self-efficacy factor were positively related to HBV vaccination intention (OR = 2.15, 95% CI: 1.25–3.71; OR = 2.75, 95% CI: 1.62–4.66) while the response costs was negatively related to the HBV vaccination motivation (OR = 0.50, 95% CI: 0.29–0.83). The socio-demographic variables showed that younger, married and good self-rated health status participants were statistically associated with the intention of taking the HBV vaccine. Sex, education level and income group were not significantly associated with vaccination intention. The migrant-industry variables showed that migrant location had a strong effect on migrant workers’ vaccination intention.

**Conclusion:**

Socio-demographic, migrant-industry variables and PMT factors (severity, self-efficacy and response costs) were statistically associated with migrant workers’ intention to vaccinate. Our results suggest that health policy makers should provide more information to migrants on HBV severity; inform migrant workers on where, when and how to get the HBV vaccine; tap into work organizations as a location for vaccinations; and identify migrant worker subgroups for targeted interventions.

## Background

The hepatitis b virus (HBV) is ranked first among class A and B statutory reported infectious diseases in China, with 60–80,000 acute HBV cases reported each year [1]. While the routine immunization of children beginning in 1992 significantly reduced the horizontal transmission of HBV, the vertical integration of HBV among adults poses a significant health challenge to China [2, 3]. Adults also accounted for most of China’s 288 million migrant workers in 2018, making up more than one third of the entire working population [4]. With the HBV transmission route running through poor living conditions and high geographical mobility, migrant workers are a highly susceptible transmitter-recipient HBV population and a vulnerable spot in China’s immunization procedures [5–7].

While the HBV vaccine can effectively prevent the spread of HBV among adults, migrant workers’ lower vaccination prevalence than permanent workers is due to their poorer cognition of HBV and the HBV vaccine, lower education level and poorer health awareness levels, living in groups and lower accessibility to health care services [8–11]. While previous Chinese studies have identified an individual’s knowledge and cognition of HBV and the HBV vaccine as key factors explaining HBV vaccination intentions and behavior, these studies only employed partial measures of HBV cognition [12–14]. One exception was a study using protection motivation theory (PMT) to specify and test a cognition model of migrant workers’ HBV vaccination intention, but the migrant-industry characteristics and key PMT factors, such as response costs, were excluded from the model [15]. Addressing these limitations, this paper applies a full PMT model, including migrant-industry variables and response costs, to explain migrant workers’ HBV vaccination intention in the Binhai high migration region of Tianjin, and in three Tianjin industry sectors (manufacturing, retail and service), with a high proportion of migrant workers.

**Fig. 1:**
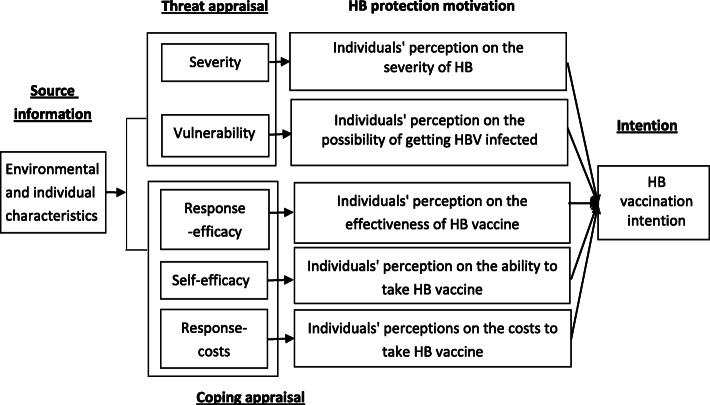
Protection motivation model for intention to take HBV vaccine of migrant workers

PMT is a widely used and powerful theoretical framework for assessing how individuals are motivated to react in a self-protective way towards a perceived health threat, which has informed public health issues in practice, especially implementing targeted health intervention strategies [16, 17]. As set out in Fig. 1, individual cognition in the PMT model is divided into three parts: information source (derived from individual and environmental characteristics), threat appraisal (severity and vulnerability) and coping appraisal (response efficacy, self-efficacy and response costs) [18, 19]. Severity refers to the individual’s subjective perception of the severity of a disease [19]. Vulnerability refers to an individual’s cognition of the likelihood of getting sick, related to an individual’s perception of the probability of being infected by HBV [20]. Response efficacy refers to the individual’s perceived effectiveness of protective behavior in preventing and controlling the HBV threat [19]. Self-efficacy is the individual’s ability to take protective action [19]. Response costs are the barriers to taking protective behaviour [19], mainly the price individuals would pay for protective behavior, measured by the cost of the HBV vaccine, but also the extent of HBV vaccination knowledge and worries about the side effects of the vaccine. Generally, the higher the individual’s perception on severity, vulnerability, response efficacy and self-efficacy, the more likely protective behaviour is taken, while response costs hinder protective motivation or behavior.

We apply a full PMT model to answer the following questions: What was the intention of Tianjin migrant workers to receive the HBV vaccine?; How did Tianjin migrant workers assess the cost-benefit calculation of paying for the HBV vaccine?; and How can migrant workers’ intentions to receive the HBV vaccine be improved? The answer to these questions can improve HBV vaccination policy in Tianjin and China.

## Methods

Binahi New District in Tianjin provides a unique region for the study of migrant workers’ HBV vaccination intention and for developing strategies to prevent and treat HBV for susceptible and high-risk migrant populations. Over the last decade, Tianjin’s migrant population grew at 12–15% each year [21], with 5 million migrants accounting for 32% of Tianjin’s population and about 60% of the Binhai New District population in 2017 [22]. Besides having a poorer cognition of HBV and the benefits of the HBV vaccine, Tianjin’s migrant workers were excluded from the free 2018 vaccination program for local workers in close contact with HBV [23]. Migrant workers faced a cost-benefit calculation on whether to pay for the HBV vaccine or not. First, our full PMT model includes Tianjin migrant workers’ response costs motivation to pay voluntarily for the HBV vaccine. Second, our model includes migrant-industry variables, specifically migrant location, whether migrants were accompanied or not by other family members and industry sector. Finally, our model includes health related behavior or intentions, mainly worker cognition, risk perceptions and likelihood to follow others in HBV vaccination intention.

### Study participants

We surveyed migrant workers, who had lived for 6 months in the Binhai New District in Tianjin, but did not possess a Binhai New District household registration hukou. Given the high mobility and the lack of local registration of migrants, sampling “hidden populations”, such as migrant workers, is difficult [24]. Employing the convenience sampling method, used, for example, in a previous study of migrant children’s immunization in Beijing [25], we surveyed three migrant worker-dense industries between July and October in 2017: (1) manufacturing (including workers from food and electronic manufacturing companies); (2) retail (including workers in supermarket and electrical appliances selling markets); and (3) services (including taxi drivers, hotel and restaurant workers). Following previous studies on sample size selection [26, 27], we calculated that at least 385 migrant workers should be surveyed based on the expected willingness to participate of 50% and a tolerance error of 0.1. Considering 10% dropped samples and balancing the sample size in each industry, questionnaires were administered to 150 workers in each industry who met the inclusion criteria of living at least 6 months in the Binhai New District in Tianjin, but did not possess a Binhai New District household registration hukou, and self-identified as having no HBV vaccination history. After excluding unqualified and incomplete questionnaires, our sample consisted of 406 migrant workers, 133 from the retail, 119 from the manufacturing and 154 from the services industry, with a response rate of 90.2%. We divided migrants into Tianjin migrants, with a non-Binhai New District hukou, but a Tianjin hukou, and non-Tianjin migrants, with a non-Binhai New District and non-Tianjin hukou.

### Data collection

A pilot survey was performed both to train postgraduate students from Tianjin University of Traditional Chinese Medicine to conduct the survey and to test the questionnaire for its comprehension. Supported by the Binhai local health authority, we contacted the principals in selected companies and factories to facilitate the survey and to ensure a high response rate. Data on participants’ socio-demographic characteristics, migrant-industry characteristics, PMT measurement items and HBV vaccination intentions were obtained in face-to-face interviews, where all participants were volunteers and informed about their right not to answer questions. The intention to take the HBV vaccine was measured by a self-report question: “Will you take the HBV vaccine in the future?” with a binary yes or no response. The vaccination intention rate was 67.2%.

### PMT measurements

Based on previous studies and migrant workers’ characteristics, we designed items for each PMT construct using a 5-point Likert scale ranging from 1 (strongly disagree) to 5 (strongly agree). The severity of HBV was measured by the migrant workers’ cognition on clinical symptoms, the costs for the individual and their family, and social discrimination. Vulnerability refers to the migrant’s perceived infection probability compared with local workers. Similar to previous studies, response efficacy was measured by the migrant workers’ perception on safety and effectiveness of HBV vaccine. Self-efficacy was measured by migrants’ attitude to the advice of nearby people, the work organization, family, friends or doctors on vaccinations [28]. Response costs are the migrant workers’ realized barriers facing the individual to adopt the protective behavior, measured by the vaccination information and worries about the side effects of the vaccine, and the price of protective behavior, measured by the cost of the HBV vaccine.

As set out in Table 1, factor analysis and Cronbach’s alpha were employed to test the validity and reliability of the PMT measurement tool. As PMT assumes a close relationship between some of the constructs, an oblique rotation specially designed to maximize the correlation among factors was selected to extract the factors [20]. Using the minimum eigenvalue criterion of 1.0, Table 1 displays the PMT factors generated by producing a factor score on five dimensions, comprising severity, vulnerability, response efficacy, self-efficacy and response costs. Each respondent’s PMT factor scores were dichotomized by the cutoff point of their mean value: high (min ≤ factor score ≤ mean) and high (mean<factor score ≤ max).

**Table 1:** PMT assessment tool and factor analysis for migrant workers

Factors and items	Loading	Variance	Cronbach’s alpha
**F1-Severity factor**			
HBV is incurable.	0.56	4.16	0.88
It is expensive to cure HBV.	0.66		
The symptom of HBV is unbearable.	0.83		
HBV would bring huge mental stress for the family.	0.84		
HBV patients and HBV carrier would suffer from social discrimination.	0.70		
**F2-Self efficacy factor**			
I would like to take HBV vaccine if people around me mostly choose to.	0.83	2.82	0.87
I would like to take HBV vaccine if it is organized by the workplace.	0.85		
I would like to take HBV vaccine if the family and friends recommend that.	0.78		
I would like to take HBV vaccine if the doctor advice that.	0.63		
**F3-Response efficacy factor**			
HBV vaccine is developed enough for common use.	0.73	1.85	0.84
Taking vaccine is an effective way to prevent HBV.	0.75		
**F4-Vulnerability factor**			
As the migrant, the risk to get HBV infected is higher than other population.	0.61	1.24	0.81
People around me getting HBV infected are more than before.	0.63		
**F5-Response costs factor**			
It is inconvenient for me to take HBV vaccine.	0.56	1.14	0.61
I do not have enough information to take HBV vaccine.	0.48		
The side effect is serious when taking HBV vaccine.	0.65		
Cumulative(%)	87.13		

### Covariates

The covariates included socio-demographic and migrant-industry characteristics of the migrant workers. Socio-demographic characteristics consisted of age, sex, education level, marital status, self-rated health and income group. Education level was divided into three groups: low (below senior high school), medium (senior high school or equivalent) and high (above senior high school). Marital status included two categories: married and unmarried (including the divorced and widow). Self-rated health was measured by the respondents’ self-assessment of their current health compared with those who were in the same age cohort and was divided into three groups: poor, medium and good. The income group variable was based on tertiles of the respondent’s average monthly income. Migrant-industry characteristics included migrant location (non-Tianjin migrant or Tianjin migrant); whether the migrant was accompanied by family or not; and industry groups (manufacturing, retail and services).

### Data analysis

We estimated the frequency of migrant workers’ intention to obtain the HBV vaccination. Severity factor, vulnerability factor, response efficacy factor, self-efficacy factor and response costs factor were produced by principal component factor analysis. Bivariate analysis of PMT factors and covariates with the dependent variable were conducted by logistic regression modeling. As set out in Models 1–3 below, socio-demographic characteristics variables, migrant-industry variables and PMT factors were added into the binary logistic regression model, where the dependent variable was the intention to HBV vaccinate.

*Model 1: f*^1^(*P*) = *α*^1^ + *β*^1^_1_*X*_**Socio−demographic characteristics**_ + *ε*^1^

*Model 2: f*^2^(*P*) = *α*^2^ + *β*^2^_1_*X*_**Socio−demographic characteristics**_ + *β*^2^_2_*X*_**Migrant**−**industry characteristics**_ + *ε*^2^

*Model 3: f*^3^(*P*) = *α*^3^ + *β*^3^_1_*X*_**Socio−demographic characteristics**_ + *β*^3^_2_*X*_**Migrant**−**industry characteristics**_ + *β*^3^_3_*X*_**PMT factors**_ + *ε*^3^

All statistical analysis was performed using STATA 15.1 with two-tailed tests and *p* < 0.05 was taken as the statistically significant level. Given the nested characteristics of the models, the likelihood ratio test was employed to show the goodness of fitness of the model.

## Results

Table 2 present the characteristics of the 406 respondents. The sex of the respondents was roughly equal (male 51.7%); 58.6% were over 35 years old; 53.7% had an education level of above senior high school; and 74.1% were accompanied by family. In our sample, 273 (67.2%) intended to take the vaccine in the future, while 133 respondents (32.8%) had no intention to vaccinate. For the PMT scores, the low severity (53.0%), response efficacy (54.4%) and self-efficacy (51.7%) groups were slightly larger than the high groups, while the response costs (60.7%) and vulnerability (57.1%) groups were larger than the low groups. HBV vaccination intentions differed by age (*p* = 0.006), self-rated health (*p* = 0.085), income (*p* = 0.053), severity (*p* = 0.003), self-efficacy (*p* = 0.000) and response costs (*p* = 0.011).

**Table 2:** Characteristics of migrant workers in Tianjin and bivariate analysis of the variables

Characteristics	N(%)	OR	95%CI	*P*
**Observations**	**406 (100)**			
**Age (years)**				
16–35	168 (41.4)	1.0		
35–45	99 (24.4)	0.74	0.43–1.28	0.284
45–65	139 (34.2)	0.51	0.32–0.83	**0.006**
**Gender**				
male	210 (51.7)	1.0		
Female	196 (48.3)	0.99	0.65–1.50	0.965
**Education level**				
Low	79 (19.5)	1.0		
Medium	109 (26.8)	1.49	0.81–2.73	0.201
High	218 (53.7)	1.39	0.82–2.38	0.222
**Marital status**				
Married	312 (76.8)	1.0		
Unmarried	94 (23.2)	1.05	0.64–1.72	0.842
**Self-rated health**				
Poor	101 (24.9)	1.0		
Medium	163 (40.1)	0.93	0.55–1.55	0.771
Good	142 (35.0)	1.63	0.94–2.84	**0.085**
**Income group**				
Low	133 (32.8)	1.0		
Medium	96 (23.6)	0.93	0.54–1.60	0.793
High	177 (43.6)	1.61	0.99–2.62	**0.053**
**Migrant location**				
Non-Tianjin migrants	49 (12.1)	1.0		
Tianjin migrants	357 (87.9)	1.12	0.59–2.14	0.733
**Migrant accompanied**				
Yes	301 (74.1)	1.0		
No	105 (25.9)	0.91	0.57–1.46	0.699
**Industry**				
Manufacturing	133 (32.8)	1.0		
Retail	119 (29.3)	1.54	0.91–2.61	0.107
Services	154 (37.9)	1.51	0.92–2.46	0.101
**Severity**				
Low	193 (53.0)	1.0		
High	171 (47.0)	1.98	1.26–3.10	**0.003**
**Vulnerability**				
Low	156 (42.9)	1.0		
High	208 (57.1)	0.98	0.63–1.52	0.923
**Response efficacy**				
Low	198 (54.4)	1.0		
High	166 (45.6)	0.85	0.55–1.32	0.464
**Self-efficacy**				
Low	188 (51.7)	1.0		
High	176 (48.3)	2.66	1.68–4.20	**0.000**
**Response costs**				
Low	143 (39.3)	1.0		
High	221 (60.7)	0.55	0.34–0.87	**0.011**

Odds ratios 95% CI and *p* value are shown

In the measurement of PMT, two items (“The number of HBV patients and carriers are larger than before” and “The price of HBV vaccine is very expensive”), whose contribution rate was less than 0.45, were screened out in the factor analysis [29]. Using the minimum eigenvalue criterion of 1.0, factor analysis yielded five conceptually distinct factors that explained 87.13% of the total variance in measuring PMT. As shown in Table 1, the overall Cronbach’s alpha coefficient for PMT was 0.84, with the Cronbach’s alpha coefficient 0.88 for severity; 0.87 for vulnerability; 0.84 for response efficacy; 0.81 for self-efficacy; and 0.61 for response costs.

The results of logistic regression are shown in Table 3. In Model 3, severity (OR = 2.15, 95% CI:1.25–3.71) and self-efficacy (OR = 2.75, 95% CI:1.62–4.66) were positively and significantly related to vaccination intention and the response costs (OR = 0.50, 95% CI:0.29–0.83) was negatively and significantly related to motivation to take the vaccine. The likelihood ratio (LR) test in Table 3 shows that the PMT constructs improved the goodness of fitness of the model (LR chi2 (5) = 33.27 and *p* < 0.05). As for the migrant-industry variables, Tianjin migrant workers were more likely to choose the vaccine in the future than non-Tianjin migrants (OR = 3.310, 95% CI:1.37–7.99), while migrants accompanied by family and industry type had no influence on vaccination intentions. Younger, married and good self-rated health status participants were statistically associated with the intention of taking the HBV vaccine.

**Table 3:** Nested logistic regression model of determinants of HB vaccinate intention among migrant workers in Tianjin

Variables	Model 1			Model 2			Model 3		
	OR	95%CI	*P*	OR	95%CI	*P*	OR	95%CI	*P*
**Gender**									
Male	1.0			1.0			1.0		
Female	0.84	0.53–1.34	0.463	0.80	0.49–1.28	0.345	0.71	0.43–1.18	0.191
**Age**									
16–35	1.0			1.0			1.0		
35–45	0.44	0.21–0.90	**0.025**	0.38	0.18–0.80	**0.011**	0.28	0.12–0.63	**0.002**
45–65	0.36	0.18–0.72	**0.004**	0.33	0.16–0.68	**0.003**	0.35	0.16–0.77	**0.009**
**Education level**									
low	1.0			1.0			1.0		
Medium	0.73	0.34–1.59	0.428	0.65	0.27–1.56	0.330	0.56	0.22–1.42	0.225
High	1.21	0.66–2.23	0.536	1.26	0.65–2.43	0.492	1.23	0.62–2.48	0.554
**Marital status**	1.81	0.93–3.54	0.080	2.29	1.10–4.80	**0.028**	3.17	1.44–6.98	**0.004**
**Self-rated health**									
Poor	1.0			1.0			1.0		
Medium	1.01	0.57–1.79	0.962	1.20	0.66–2.19	0.553	1.78	0.91–3.46	0.090
Good	1.88	1.00–3.54	0.052	2.17	1.10–4.25	**0.025**	3.37	1.61–7.09	**0.001**
**Income group**									
Low	1.0			1.0			1.0		
Medium	0.63	0.35–1.14	0.129	0.60	0.33–1.12	0.107	0.65	0.34–1.24	0.191
High	1.21	0.70–2.10	0.497	1.41	0.80–2.48	0.236	1.26	0.68–2.34	0.466
**Migrant location**									
Tianjin migrant				1.0			1.0		
Non-tianjin migrant				2.55	1.15–5.65	**0.021**	3.31	1.37–7.99	**0.008**
**Migrant accompanied**									
Yes				1.37	0.79–2.38	0.264	1.27	0.71–2.28	0.428
No				1.0			1.0		
**Industry**									
Manufacturing				0.61	0.32–1.17	0.137	0.68	0.34–1.38	0.287
Retail				1.0			1.0		
Service				1.11	0.56–2.19	0.760	1.26	0.62–2.58	0.521
**Severity**									
Low							1.0		
High							2.15	1.25–3.71	**0.006**
**Vulnerability**									
Low							1.0		
High							1.07	0.64–1.78	0.809
**Response efficacy**									
Low							1.0		
High							0.86	0.50–1.46	0.565
**Self-efficacy**									
Low							1.0		
High							2.75	1.62–4.66	**0.000**
**Response costs**									
Low							1.0		
High							0.50	0.29–0.83	**0.008**
**Pseudo R2**	0.050			0.074			0.146		
**P**	0.011			0.002			0.000		
**-2LL**	438.56			427.48			394.21		

Odds ratios 95% CI and *p* value are shown

## Discussion

Based on PMT, our study investigated the association between protection motivation and HBV vaccination intention among migrant workers in Tianjin, China. To our knowledge, this is the first attempt to integrate all PMT constructs into a HBV vaccination intention study for a migrant population. We found that a significant percentage of the migrant workers, 133 of the respondents or 32.8% of the sample, lacked the intention to vaccinate and that HBV protection motivation strongly affected migrant workers’ HBV vaccination intention. Given that migrant workers are a susceptible population to HBV and a vulnerable spot in China’s immunization procedures, our study recommends specific health education designs and immunization management polices as key interventions for addressing HBV transmission.

As several PMT meta-analyses [16, 17] argued, the specificity of the PMT measurement for special populations are crucial, especially when PMT-based research is used to guide health policy at an operational level. Validating our PMT approach, Model 3 shows that severity, self-efficacy and response costs were significant constructs determining migrant workers’ HBV vaccination intention. While our results share similarities with other PMT studies, a meta-analysis of PMT literature found that not all PMT variables are able to predict a given behavior or intention with the same strength. The role and influence of the PMT variables vary across different vaccination domains and research populations [16, 17]. For example, compared to severity and vulnerability, response efficacy, self-efficacy and response costs usually have stronger relationships with the adaptive intention to vaccinate [16]. One of the most researched areas, influenza vaccination intention studies, found that response efficacy and self-efficacy tended to be significant vaccination predictors [30, 31]. In Liu et al’s [15] partial PMT model, vulnerability and response efficacy were found to be the most significant PMT constructs determining migrants’ HBV vaccination intention. These different results reflect different study places, different measurement tools and different model specifications. Importantly, our fully specified PMT model included response costs data, information on the migration location, and whether the migrant was accompanied by family members and industry covariates.

The effect of severity in our PMT model of vaccination intention suggests that the content of HBV education messages should emphasize the severity of HBV, including identifying symptoms, the heavy economic burden, the worries and pain on family members, the barriers to acquiring employment and potential social discrimination. Self-efficacy as a strong predictor of migrant workers’ vaccination intention informs health authorities to tap into work organizations as a location for vaccinations where other workers being vaccinated encourages individuals to vaccinate. According to the response costs content, barriers to migrant workers’ vaccinations should be addressed directly by health education content and vaccination management policy, including side effects of the vaccine. Health authorities should provide more information on where, when and how to get the HBV vaccine and establish more vaccination sites, especially in migrant workers’ workplaces or by providing visiting injection services.

For the socio-demographic and migrant-industry variables, marital status, self-rated health and migrant location were the key determinants to the migrant workers’ vaccination intention, which suggests that HBV prevention and education policy should focus on the migrant workers who are in the older, unmarried and in poor self-rated health status groups and HBV health education and vaccination management policy should concentrate on migrant workers from other provinces. We expected industry differences since migrant workers in the retail and service industry were required by the government to undergo a physical examination before starting work. This absence of industry differences might reflect a high level of inadequate HBV and HBV vaccine knowledge by migrant workers irrespective of industry sector. If migrant workers were ill-informed about HBV, then health officials are missing an opportunity to educate migrant workers in services and retail industries on the benefits of the HBV vaccine during the physical examination process. One place to improve migrant worker HBV education is during the existing testing of workers. We also recommend an expansion of the testing regime to all workplaces.

### Study limitations

Maladaptive response rewards were not included in our PMT model. In some previous vaccination studies, maladaptive response rewards were measured by saving money or time, avoiding the side effects of the vaccine and acquiring natural immunity to subsequent infection [31, 32]. Since HBV is incurable, acquiring natural immunity does not exist, and saving expenses and worries about the side effects have been included in response costs, so maladaptive response rewards may not be a serious omission in our PMT model. However, maladaptive response rewards warrants empirical study in future HBV research. Second, our study did not assess vaccination behaviour directly. While it has been shown that vaccination behavior in empirical studies can be predicted by previous intention in a wide range of contexts [33, 34], future studies should include vaccination behavior directly. Third, as a result of convenience sampling, our results and findings need confirmation through studies of other migrant worker populations, regions and industries. Finally, data on Tianjin’s migrant workers was collected through a convenience sample, which may limit the external generalisation of our results.

## Conclusion

Our results showed that severity, self-efficacy and response costs from PMT were statistically associated with migrant workers’ intention to vaccinate in Tianjin, China. The significance of these three PMT constructs inform health policy makers on how to improve health education and immunization policy for migrant workers’ HBV vaccination intentions. We recommend policy makers provide more information to migrants on HBV severity; inform migrant workers on where, when and how to get the HBV vaccine; tap into work organizations as a location for vaccination; and identify migrant worker subgroups for targeted interventions.

## Data Availability

The datasets used and analysed during the current study are available from the corresponding author on reasonable request.

## Abbreviations

HB: Hepatitis B
HBV: Hepatitis B Virus
PMT: Protection Motivation Theory
